# Tools for genetic manipulation of the endemic fungal pathogen *Emergomyces africanus* and application of a fluorescent reporter strain in infection models

**DOI:** 10.1128/msphere.00180-26

**Published:** 2026-07-02

**Authors:** Lucian Duvenage, Alisha Chetty, Darren D. Thomson, Elizabeth R. Ballou, Nelesh P. Govender, Chad A. Rappleye, J. Claire Hoving

**Affiliations:** 1 CMM AFRICA Medical Mycology Research Unit, Institute of Infectious Disease and Molecular Medicine, Faculty of Health Sciences, University of Cape Town 63726, Cape Town, South Africa; 2 Wellcome Centre for Infectious Diseases Research in Africa, Institute of Infectious Diseases and Molecular Medicine, University of Cape Town, Cape Town, South Africa; 3 Division of Immunology, Department of Pathology, Faculty of Health Sciences, University of Cape Town 63726, Cape Town, South Africa; 4 MRC Centre for Medical Mycology, The University of Exeter, Exeter, United Kingdom; 5 Wits Mycology Division, School of Pathology, Faculty of Health Sciences, University of the Witwatersrand 318041, Parktown, Gauteng, South Africa; 6 National Institute for Communicable Diseases, a division of the National Health Laboratory Service, Johannesburg, Gauteng, South Africa; 7 Department of Microbiology, The Ohio State University 215854, Columbus, Ohio, USA; University of Guelph, Guelph, Ontario, Canada

**Keywords:** mycology, molecular methods, animal models, *Emergomyces africanus*

## Abstract

*Emergomyces africanus* is a thermally dimorphic fungal pathogen endemic to Southern Africa, which can cause fatal systemic infections in persons with advanced HIV disease. Its mechanisms of pathogenesis are not well understood. Characterization of virulence traits in this pathogen requires appropriate molecular tools for genetic manipulation. Molecular technologies developed for the transformation of *Histoplasma capsulatum* were adapted for use in *E. africanus. Agrobacterium*-mediated transformation was used to generate a reporter strain expressing green fluorescent protein (GFP). The *E. africanus* GFP reporter strain facilitated the study of yeast interaction with macrophages *in vitro* and allowed the identification of infected phagocyte cell types in the mouse lung by flow cytometry. *E. africanus* could also maintain episomal plasmids with telomere-like sequences to introduce expression constructs without genome modification. Using this plasmid system, RNA interference constructs were used to knock down the expression of cell wall α(1,3)-glucan by targeting the transcripts of the α-glucan synthase (*AGS1*). An episomal CRISPR/Cas9 system was evaluated for *E. africanus*, which effectively disrupted *GFP* in a reporter strain and enabled the generation of a *URA5* uracil auxotroph. These tools and strains will facilitate future studies to elucidate the mechanisms of pathogenesis of *E. africanus*.

**IMPORTANCE:**

*Emergomyces africanus* is an opportunistic fungal pathogen affecting persons with advanced HIV disease in South Africa. The biology and pathogenesis of *E. africanus* are not well understood, as the importance of the disease caused by this fungus (emergomycosis) has only been recognized in recent years, and molecular studies have been impaired by the lack of genetic technologies. In this work, we describe tools and methods for the genetic modification of this pathogen, which will accelerate future studies investigating how the fungus causes disease in the human host. These essential tools include (i) the ability to create fluorescent reporter strains, such as the green fluorescent protein *E. africanus* strain described here, which facilitates tracking the spread of the fungus during infection and enhances microscopy studies, (ii) methods for knocking down gene expression in *E. africanus*, and (iii) the permanent disruption of genes through CRISPR/Cas9 gene editing.

## INTRODUCTION

*Emergomyces africanus*, part of the recently classified genus *Emergomyces* (1), is a fungal pathogen of humans endemic to Southern Africa (2). The fungus can cause life-threatening systemic infection in immunocompromised individuals, which, in South Africa, has exclusively been in persons with advanced HIV disease (3). Because this pathogen is less often suspected by clinicians, patients affected by emergomycosis are often diagnosed only at the late stages of infection, characterized by widespread skin lesions. An understanding of its biology and host-pathogen interaction is needed to develop effective diagnostics and treatment strategies for this infection in high-risk populations.

*E. africanus* is a thermally dimorphic fungus. It exists in a saprobic mycelial phase in soil and releases airborne conidia (4), which, when inhaled by the host, transition to a yeast phase triggered by body temperature (5). The mechanisms by which these yeasts infect host cells are unknown. The ability to genetically manipulate a fungal pathogen can open new avenues of research, whether by examining the effects of gene deletions or introducing reporter genes for a wide range of applications, such as facilitating the detection of the pathogen in infection models. New tools or protocols must be developed or adapted for non-model fungi such as *E. africanus*. In this study, we used molecular tools originally developed for *Histoplasma capsulatum* and other dimorphic fungi (6–11) and adapted them to the genetic modification of *E. africanus*.

In some fungi, notably the dimorphic fungal pathogens, homologous recombination is inefficient, which prevents gene replacement strategies. In these cases, *Agrobacterium-*mediated gene transfer (ATMT) has been employed to introduce genes of interest (9, 11) or fluorescent transcriptional reporters, or to create random insertion libraries for phenotypic screening. Co-culture of *Agrobacterium tumefaciens*, harboring the shuttle plasmid, with the fungus results in the insertion of a single copy (in the majority of cases) of the T-DNA randomly in the genome (12). This method has been used successfully for several of the thermally dimorphic pathogens, including *Histoplasma capsulatum*, *Blastomyces dermatitidis*, *Paracoccidioides brasiliensis*, and *Talaromyces marneffei* (as reviewed in reference 13). We used ATMT to introduce a green fluorescent protein (GFP) transgene into *E. africanus*, using shuttle vectors and a protocol previously developed for the transformation of *H. capsulatum* (14). The *E. africanus* GFP reporter strain facilitated imaging of yeast-macrophage interactions and identification of phagocyte cell types in the lung associated with the yeast in the mouse model of infection (15) by flow cytometry analysis.

Shuttle vectors which replicate in *E. coli* can be maintained by *H. capsulatum* as linear plasmids through the addition of telomere sequences (16–18). These vectors are useful for introducing expression constructs without genome modification. For example, RNA interference (RNAi) constructs can be used to knock down expression of virulence-associated genes (7, 18–22). These plasmids are rapidly lost without selection pressure, which is useful when transient expression is desired, e.g., expression of the genome-modifying enzyme Cas9. Gene deletion/mutation is a powerful tool to study putative virulence-associated traits. CRISPR-Cas9 genome editing has been applied to study these traits in thermally pathogenic fungi, including *Blastomyces dermatitidis* (23) and *Histoplasma capsulatum* (6, 24). In this study, we applied an episomal plasmid system designed for *H. capsulatum* for CRISPR-Cas9 genome editing (6) in *E. africanus* to delete distinct genes in the genome.

With effective molecular genetic tools in place, and their application optimized for *E. africanus*, its virulence traits and pathogenesis can be investigated in future work. This knowledge could facilitate the development of more specific diagnostic tests for early screening of at-risk patients or inform clinical management of the disease.

## RESULTS AND DISCUSSION

### Generation of fluorescent strains using *Agrobacterium*-mediated transformation of a GFP transgene

**Fig 1:**
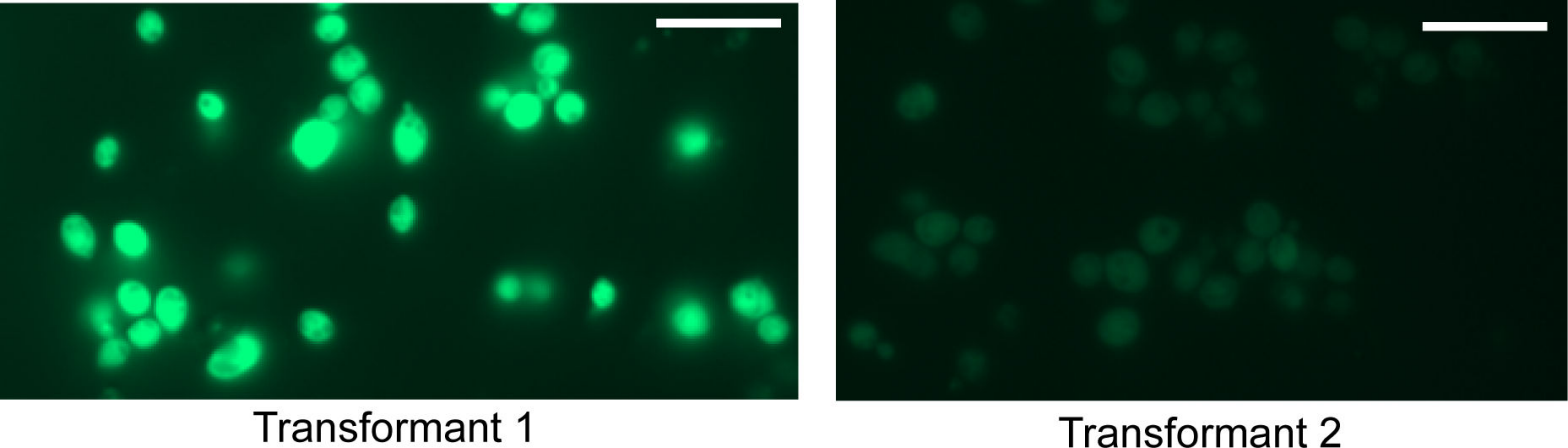
GFP-expressing *E. africanus* derived through ATMT. *E. africanus* GFP-expressing transformants were derived by transformation with *A. tumefaciens* harboring pAG22. Individual transformant colonies were evaluated by fluorescence microscopy, showing variable expression levels of GFP between transformants under constant imaging conditions. Bar = 10 µm.

We used *Agrobacterium tumefaciens-*mediated transformation (ATMT) to transform *E. africanus* using the shuttle vector pAG22, which encodes a GFP transgene. GFP and hygromycin phosphotransferase are expressed under the control of *H. capsulatum* regulatory elements: *TEF1* (translation elongation factor) constitutive promoter and *TUB2 (*tubulin) terminator, and *RPL1B* (large ribosomal subunit protein) promoter and *RPL7* (large ribosomal subunit protein) terminator, respectively (Fig. S1). Transformation of *E. africanus* using *A. tumefaciens* LBA1100 or EHA105 strains carrying pAG22 yielded similar numbers of transformants. Following transformation, individual transformants were screened for GFP expression levels, as insertion of the T-DNA in different loci can result in varying levels of GFP expression, and, although uncommon, multiple insertions of the T-DNA can occur (12). To identify reporter strains with high GFP expression, transformant colonies were grown in liquid BHI broth and inspected by fluorescence microscopy. Figure 1 shows an example of differences in GFP fluorescence levels between two transformants.

One pAG22 transformant with high fluorescence was selected for further applications. In preliminary experiments with the mouse model of infection, it was noted that the virulence of laboratory-passaged *E. africanus* (including untransformed wild-type strains) was reduced, as higher doses of yeast were required to replicate pathology compared to previous work. Therefore, all wild-type and control strains used in this study were passaged under the same conditions in the laboratory as would be required to generate respective transformants. An intranasal dose of 1 × 10^7^ yeast cells was then used to infect mice, as this could replicate the pathology seen in previous work (15). The GFP-fluorescent strain retained pathogenesis compared to the passage-matched wild-type strain in survival studies (Fig. S2).

### Fluorescent *E. africanus* yeast interaction with macrophages

**Fig 2:**
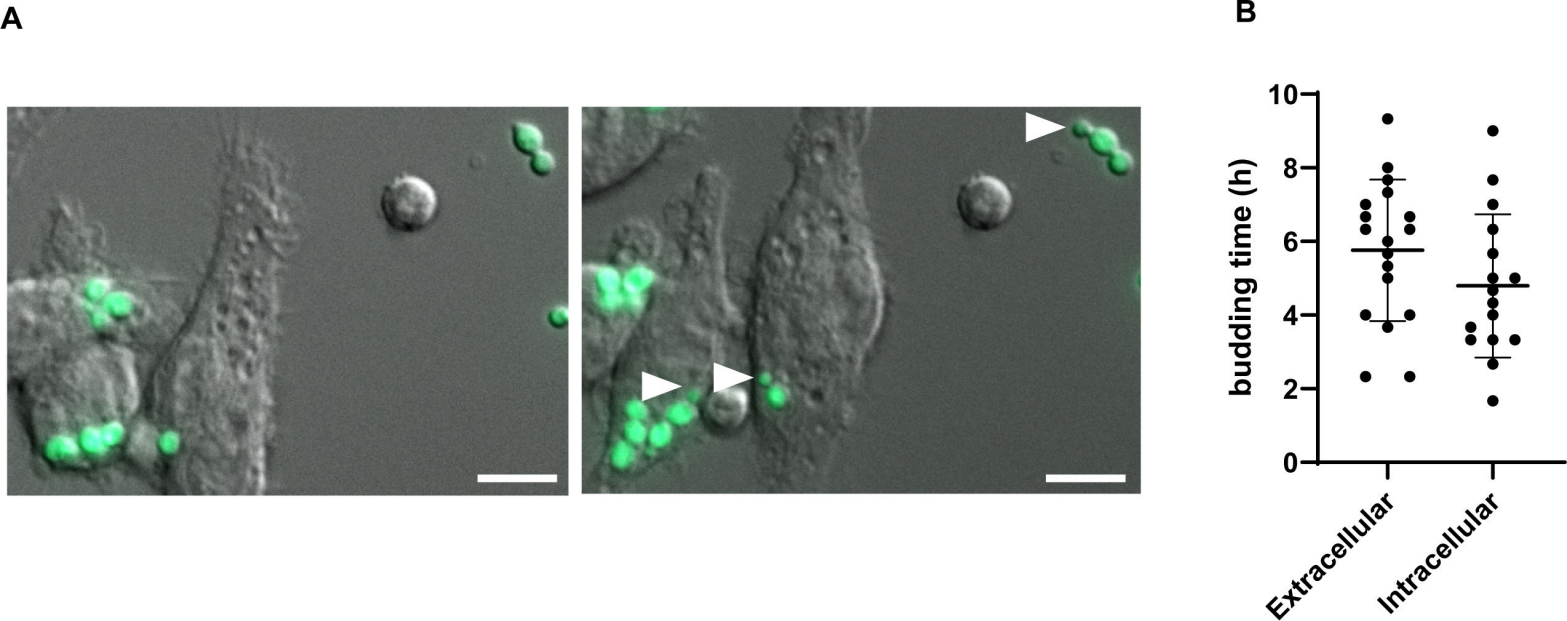
An *E. africanus* GFP-fluorescent reporter strain facilitates the study of intracellular yeast growth. GFP-fluorescent reporter yeast was added to J774.1 macrophage cells in chambered imaging slides. Time-lapse imaging was started 1 h after yeast addition (*t* = 0), at which point several yeast cells had been internalized (a representative example is shown in panel **A**, left). The emergence of new buds (panel **A**, right, indicated by arrowheads) was recorded. (**B**) Observed budding time was summarized for several fields of view that contained a mix of both extracellular and intracellular yeasts, *P* > 0.05 (mean ± SD). Bar = 10 µm.

The GFP-fluorescent yeast facilitated imaging of *E. africanus* interaction with macrophages. J774A.1 macrophages in chambered imaging slides were infected with *E. africanus* yeast (MOI = 1) and imaged every 20 min. Un-opsonized yeast cells are readily phagocytosed, and a high proportion of yeast cells were intracellular at the start of imaging, 1 h following yeast addition. Intracellular yeast could easily be observed by their GFP fluorescence (Fig. 2A). Internalization of yeast was confirmed by examination of Z-stack data. Budding of intracellular yeast could be measured in the time-lapse data, demonstrating phagocytosed *E. africanus* yeasts remained viable. The observed budding time of intracellular yeast was not significantly different from that of extracellular yeast in the same proximity (Fig. 2B). Yeast cells continued to proliferate intracellularly over time, with some macrophages appearing heavily infected and unable to control yeast proliferation. These data indicate that the intracellular niche is permissive to *E. africanus* replication, as it is for *H. capsulatum*. Previous work showed that bone marrow-derived macrophages are not actively lysed by *E. africanus*, unlike *H. capsulatum* (25). These data suggest that, similar to *H. capsulatum* and *P. brasiliensis*, non-activated phagocytes are unable to control *E. africanus*, which may aid in fungal systemic dissemination during infection (26, 27). Further studies using the GFP reporter strain and live cell imaging could provide more information on the dynamics of intracellular replication and macrophage cell death.

### Identification of infected phagocyte populations in the mouse lung following infection with the GFP reporter strain

**Fig 3:**
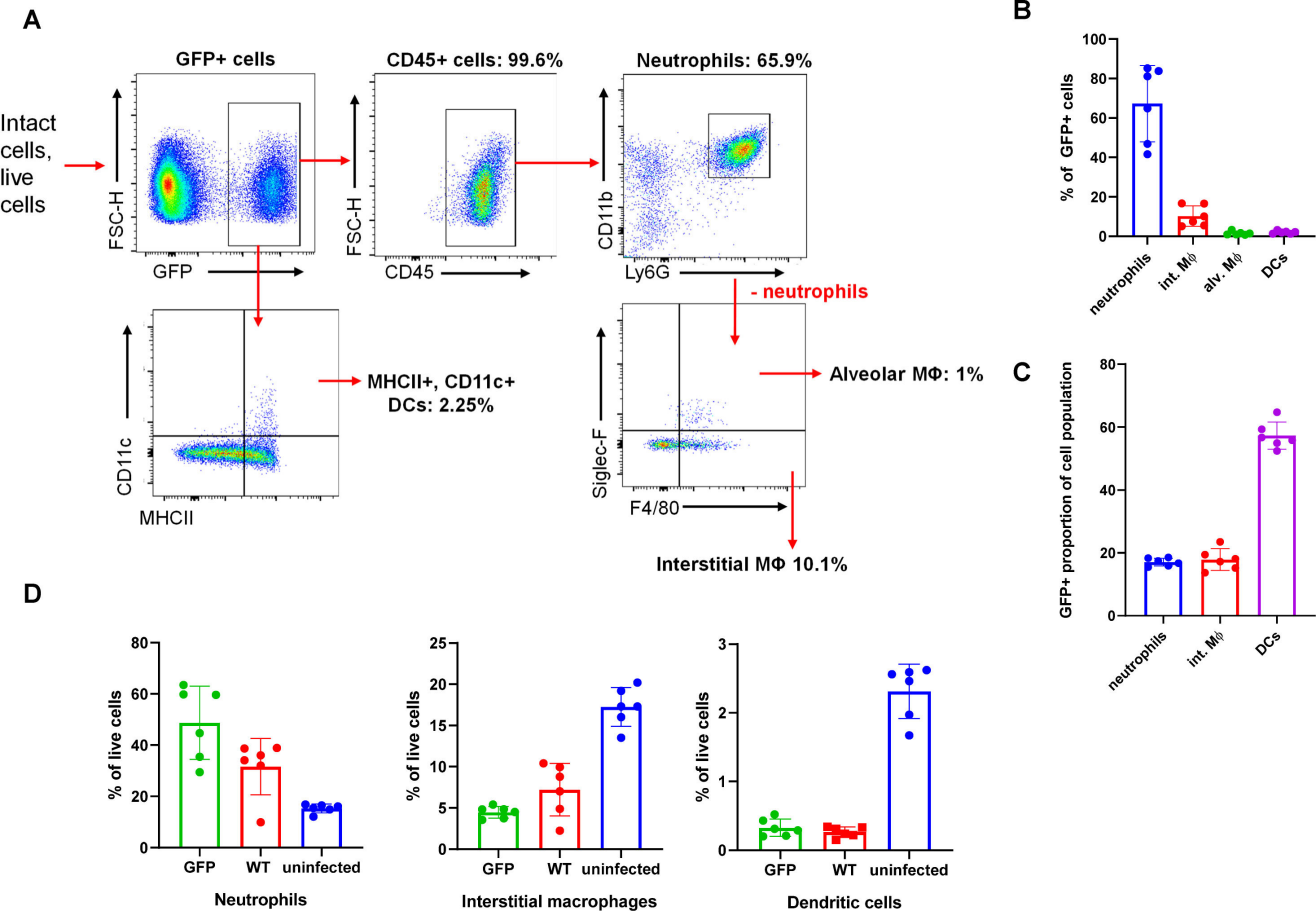
Identification of myeloid cell types associated with GFP-expressing *E. africanus* in mouse lung at 7 days post-infection. Scatter plots showing the strategy of gating on GFP first (**A**), which was used to identify myeloid cell types in the mouse lung that were GFP-positive (associated with the GFP reporter yeast), as summarized in panel **B**. (**C**) Alternate gating on the various cell types first determined the proportions of each cell type associated with GFP yeast. (**D**) Myeloid cell distribution in the WT and GFP strain-infected mouse lung and in uninfected mouse lung. Differences in cell recruitment between WT- and GFP-strain-infected mice were not significant, *P* > 0.05 (mean ± SD). *n* = 6 mice per group, data are representative of two independent experiments.

We applied the GFP-fluorescent *E. africanus* strain to examine its association with phagocytes *in vivo*. Mice were infected intranasally with the fluorescent *E. africanus* strain, and after 7 days, myeloid cell recruitment in the lungs was analyzed by flow cytometry. Lungs from uninfected mice were analyzed as a control. Gating first on GFP+ cells (i.e., cells with internalized *E. africanus*) showed that <1% of GFP+ cells were CD45-negative (Fig. 3A), suggesting that nearly all phagocytosed *E. africanus* yeasts resided in CD45+ phagocytes. Similar results were reported in a study with an *H. capsulatum* GFP-expressing strain (28), suggesting that both pathogens persist well within this intracellular niche *in vivo*. However, these results only reflect the intracellular fungal burden at the day 7 timepoint and do not account for fungi that may have been cleared by this time. Most GFP-fluorescent yeasts were associated with neutrophils (CD11b^hi^, Ly6G^hi^) (66.4% ± 17.4% of GFP+ cells; Fig. 3B). The remaining cell types that contained GFP yeast were interstitial macrophages (10.3% ± 5.3%), dendritic cells (MHCII^hi^, CD11c+) (2.1% ± 0.7%), and a very small subset of CD11b−, CD11c− cells (Fig. 3B).

Alternatively, gating first on all live host cells showed what proportion of total cells were infected with *E. africanus* (GFP+). Of CD45+ cells within the lung, approximately 16.4% ± 4% were infected (GFP+). We then characterized the GFP-fluorescent (i.e., infected) proportion of each myeloid cell type, using a similar gating strategy. The mean proportion of DCs that were infected (GFP-positive) was 57.3% ± 4.3% of DCs detected (MHCII^hi^, CD11c^hi^), with smaller proportions of total lung macrophages and neutrophils infected (Fig. 3C). Similar proportions were reported for *H. capsulatum* with the distribution of GFP-fluorescent *H. capsulatum*-infected phagocytes at day 7 post-infection: macrophages: 25.1%, PMNs: 51.2%, and DCs: 24.3% for the G217B strain (28). DCs are pivotal in the adaptive immune response to fungi, including *H. capsulatum* (29, 30). The high proportion of DCs infected with *E. africanus* at the 7-day timepoint suggests the initiation of adaptive immunity. The cellular recruitment (of CD45+ cells) to the lung in response to the GFP-fluorescent strain was not significantly different from CD45+ cell populations in animals infected with the non-fluorescent wild-type control (Fig. 3D), indicating the GFP-fluorescent *E. africanus* strain stimulates a similar host immune response. Previous work examined myeloid cell recruitment in *E. africanus*-infected mouse lung at 14 days post-infection, although a lower dose of yeast was used for infection (15). A time course of infection was not performed in the current study; this could be included in future experiments to gain more information on cell recruitment and infected phagocyte proportions over time.

SiglecF+ alveolar macrophages were detected in lungs from uninfected mice (1.5%–4% of total cells), but the proportion was reduced in infected mice (0.01%–0.16% of total cells). Similarly, the number of interstitial macrophages and dendritic cells was higher in uninfected mice (Fig. 3D). This could be due to the proportional increase in neutrophils recruited or apoptosis of leukocytes in the lung, a known feature of infection with *H. capsulatum* and a critical element of protective immunity. Similar studies of *H. capsulatum* infection show that T cells specifically constitute the dominant apoptotic population (31). As T-cell staining was not included in our experimental design, future studies can determine whether *E. africanus* induces a similar pattern of apoptosis in the lung.

### Transformation of *E. africanus* with episomal vectors and reduction of cell wall α(1,3)-glucan by RNA interference

*E. africanus* could be transformed with episomal plasmids based on pCR115 (18) for which linear plasmids with telomeres are maintained without chromosome integration. This was first tested by constructing a modified plasmid with hygromycin B selection (pHygR-GFP). The number of colonies obtained using electroporation of episomal plasmids was typically lower than for ATMT. Hygromycin selection pressure was maintained when working with these transformants, as these plasmids are rapidly lost from the cell population without selection.

To silence production of α(1,3)-glucan in *E. africanus*, we expressed an AGS1-targeting RNA hairpin from an episomal plasmid based on the AGS1-RNAi plasmid pCR115. The RNA hairpin is designed for RNAi-induced knockdown of the *H. capsulatum AGS1* gene, with homology to a 678 base pair region of exon 3 of this gene (18). This region is 84% similar overall in *H. capsulatum* and *E. africanus*, with several continuous sections of at least 20 nucleotides or more with 100% identity. Therefore, we hypothesized that effective RNAi silencing using the same RNA hairpin could be achieved in *E. africanus*, given that several 21–22 small interfering RNA molecules with complete sequence match are predicted to be produced following Dicer processing of the long double-stranded RNA. We replaced the GFP in pHygR-GFP with the *AGS1* inverted repeat sequence from pCR115 and used this plasmid (pHygR-HcAGS1-RNAi) to transform *E. africanus*. Yeast transformed with pHygR-HcAGS1-RNAi did not differ in growth rate compared to control yeast, which was transformed with a similar plasmid containing a GFP inverted repeat.

**Fig 4:**
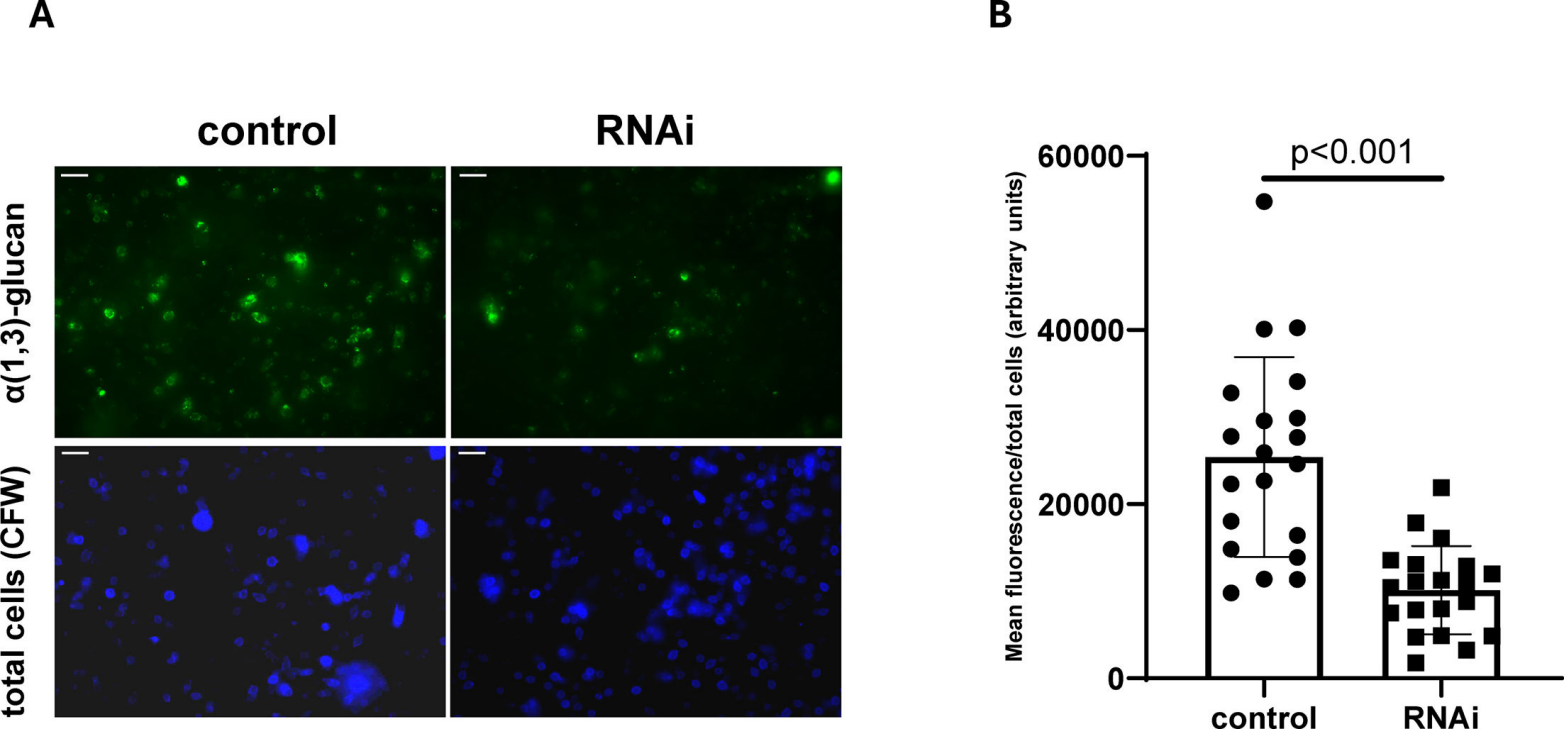
(**A**) Cell wall staining and fluorescence microscopy of wild-type and AGS1 RNAi strains of *E. africanus*. *E. africanus* harboring the AGS1 RNAi plasmid or the control GFP RNAi plasmid were stained with a monoclonal antibody specific for α(1,3)-glucan and calcofluor white (CFW) and examined by fluorescence microscopy. Imaging settings were kept constant for all samples. Bar = 10 µm. (**B**) Background-corrected mean fluorescence intensity of microscopy images was measured and normalized by the number of cells per image. Ten images each from two independent experiments were analyzed, with each filled circle (control) or square (RNAi) representing an individual measurement (mean ± SD), Student’s unpaired *t*-test, *P* < 0.001.

Reduction of cell wall α(1,3)-glucan through RNA interference was assessed by staining the AGS1-RNAi plasmid-transformed yeasts with a monoclonal antibody recognizing α(1,3)-glucan. It was noted that not all yeast cells were uniformly stained (Fig. 4A), suggesting variable amounts of α(1,3)-glucan. *H. capsulatum* also displays variation in cell wall α(1,3)-glucan levels, as assessed by staining with the same antibody during broth culture growth (32). Similarly, *E. africanus* cell wall α(1,3)-glucan levels may increase at different time points during culture or in different growth media. Nevertheless, yeasts transformed with the *AGS1* RNAi plasmid showed an overall lower level of cell wall α(1,3)-glucan staining in the population compared to the control (Fig. 4A and B). RNAi-induced gene silencing does not typically lead to a complete knockdown of the target, and due to variability in dsRNA expression, a small proportion of cells do not exhibit the full RNAi effect (18). Therefore, some α(1,3)-glucan staining was still observed in *E. africanus* yeasts maintaining the RNAi plasmid.

### Gene mutation in *E. africanus* using an episomal CRISPR/Cas9 system

**Fig 5:**
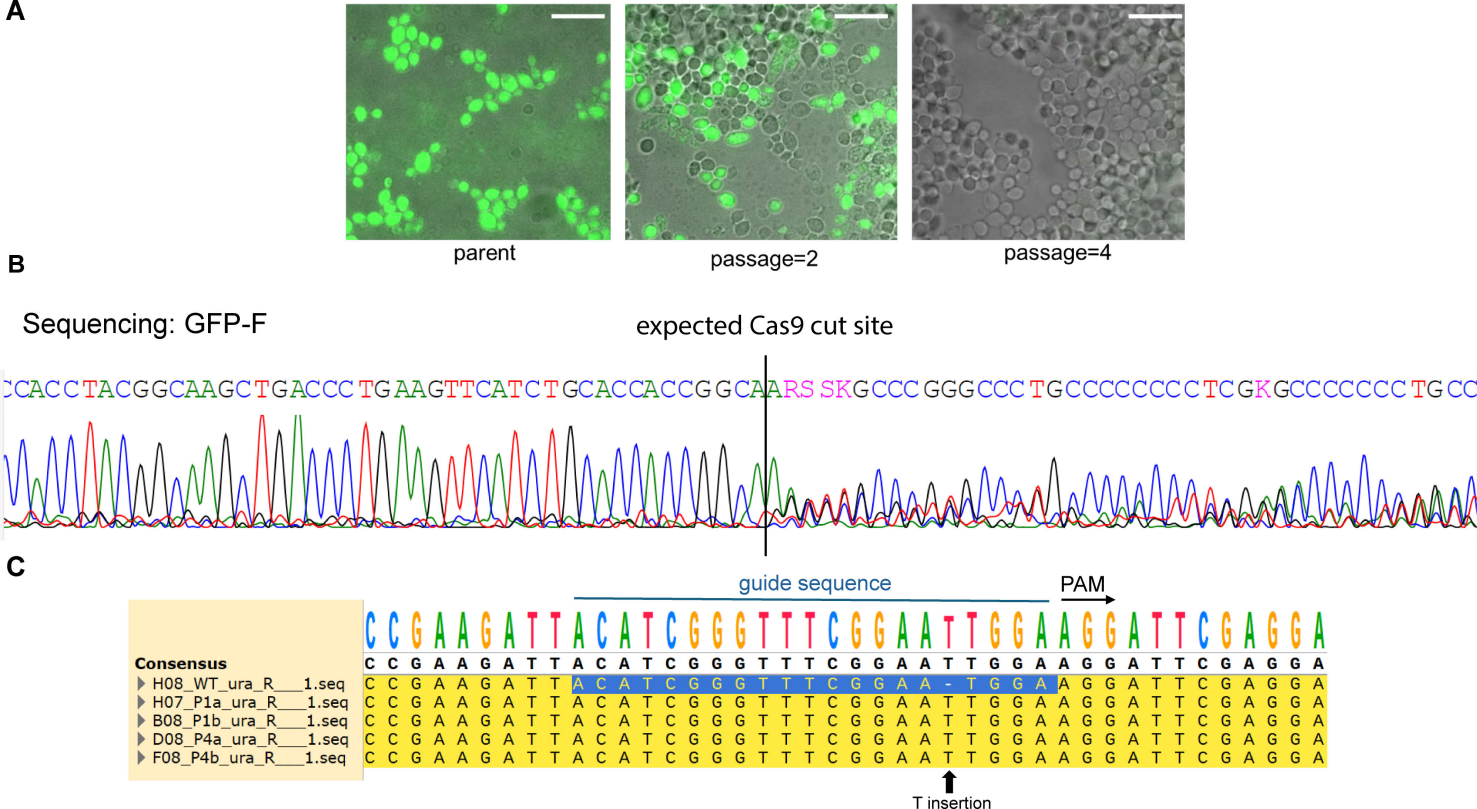
Evaluation of a CRISPR/Cas9 episomal system in *E. africanus*. (**A**) Merged fluorescence microscopy and brightfield images showing a decline in the proportion of fluorescent yeast cells with subsequent passages following transformation of GFP-expressing *E. africanus* with the CRISPR/Cas9 plasmid pSL01-sgGFP. Bar = 10 µm. (**B**) Sanger sequencing of a mixed population showed a mixture of signals following the expected Cas9 cut site, indicating the presence of various indels in the population. (**C**) Sequencing data of individually selected clones, aligned with the WT strain, following CRISPR/Cas9-mediated gene disruption of URA5. A T-insertion was observed in all clones.

To test whether CRISPR-Cas9 gene editing could facilitate gene mutation in *E. africanus*, we transformed another GFP-expressing strain of *E. africanus* with a linear plasmid expressing Cas9 and a GFP-targeting CRISPR gRNA. This GFP-fluorescent *E. africanus* strain was generated through ATMT using pAG21 (a GFP expression plasmid equivalent to pAG22, but with G418 selection). The CRISPR-Cas9 plasmid conferred hygromycin resistance and expressed a gRNA targeting the GFP gene. Hygromycin-resistant transformant colonies were passaged in BHI broth with hygromycin B selection, and the yeasts were examined by fluorescence microscopy for loss of the GFP fluorescence. After successive passages, an increasing proportion of the population was no longer GFP-fluorescent (Fig. 5A). Sequencing of the GFP gene in the transformant population confirmed a mixed population with indels at the expected Cas9 cut site (Fig. 5B), showing efficient CRISPR/Cas9-based genome editing in *E. africanus*.

Auxotroph strains are useful for genetic manipulations because they facilitate the selection of plasmids carrying genes that rescue the auxotrophy. A previous CRISPR/Cas9 workflow used for *H. capsulatum* (6) was used to disrupt *URA5* in *E. africanus* to generate a uracil auxotroph strain. The *E. africanus URA5* homolog (gene: ACJ72_02173) was identified through a BLAST search. The protein sequence is highly similar to that of *H. capsulatum* (88.1% identity, 93.0% similarity as determined by pairwise alignment using EMBOSS Water [33]). A guide RNA sequence was designed using CRISPOR (https://crispor.gi.ucsc.edu/) (34) to maximize the chance of indel generation in the *URA5* gene while minimizing off-target effects. Following transformation of wild-type *E. africanus* with the URA5-targeting CRISPR-Cas9 plasmid, followed by four passages under hygromycin selection, uracil auxotrophs were selected using 5-FOA-resistance screening. Several 5-FOA-resistant clones were identified, and their inability to grow without uracil was validated by failure to grow in media lacking uracil. Sequencing of four clones showed a single nucleotide T insertion in all clones (Fig. 5C), causing a frameshift mutation, preventing production of a functional *URA5* gene product. This *E. africanus ura5* mutant can now be used as the host for *URA5-*based expression vectors or RNAi vectors. *H. capsulatum* uracil auxotrophs are avirulent in mice, but those transformed with complementation vectors show continued virulence, suggesting that these vectors are stable *in vivo* (35). This is likely to be the case for *E. africanus* as well.

### Conclusions

In this study, we demonstrate that molecular tools developed for genetic modification of *H. capsulatum* could be adapted for use in *E. africanus*, suggesting that the two species use similar regulatory elements for high-level gene expression. Our results include the use of ATMT to transform *E. africanus* with expression plasmids, generating a GFP-fluorescent strain that can be used for tracking infections both in cultured macrophages and *in vivo*. In addition, we provide results showing the efficacy of episomal plasmids designed to induce RNA-interference-based gene knockdown and CRISPR-Cas9-based mutation of target genes. Thus, by adapting technologies, we have put into place tools and methodologies to functionally study genes in *E. africanus* through expression as well as loss-of-function. Furthermore, we have created an *E. africanus* uracil auxotroph strain enabling the use of URA5*-*based complementation vectors instead of hygromycin selection. Such URA5-based vectors have the advantage of providing for selection *in vivo*, which cannot be done with hygromycin. Together, these tools make *E. africanus* amenable to genetic modification to investigate virulence traits and how they influence the host-pathogen interaction.

## MATERIALS AND METHODS

### *E. africanus* and *A. tumefaciens* strains and growth conditions

*Emergomyces africanus* (clinical isolate CBS 136260 [4]) was grown in brain-heart infusion (BHI) broth (cat. no. 110493, Merck, USA), at 37°C, 180 rpm, or maintained on BHI agar plates (cat. no. 70138, Merck) at 37°C. *A. tumefaciens* strains LBA1100 and EHA105 were maintained on LB agar plates at 25°C. Transformation of *Agrobacterium* binary vectors into *A. tumefaciens* was done by electroporation as described in reference 36, and transformants were selected with 100 μg/mL kanamycin.

### Transformation using *Agrobacterium tumefaciens*

Transformation of *E. africanus* yeast by *Agrobacterium* was done using previously established methods for *H. capsulatum* (14), with the exception that BHI broth or BHI agar plates were used instead of *Histoplasma*-specific growth medium. *Agrobacterium tumefaciens* strains LBA1100 and EHA105 were used to transform *E. africanus* with pAG22 (GFP expression, hygromycin resistance), yielding similar results. BHI was inoculated with a colony of *E. africanus* from a fresh streak plate, and the culture was grown for 3 days at 37°C with shaking. Colonies from a streak plate of *A. tumefaciens* were used to inoculate 5 mL minimal glucose medium (14) for 24 h at 25°C. Subsequently, 1 mL of this culture was used to inoculate 5 mL induction medium containing 0.1 mM acetosyringone (Sigma-Aldrich) for further 16 h growth. *E. africanus* yeast cells were washed with PBS by centrifugation and resuspension, then resuspended in induction medium. The optical density (600 nm) of both *E. africanus* and *A. tumefaciens* suspensions was determined, then combined in 900 µL to give final optical densities of 1.0 for each. Suspensions with an OD600 of 1.0 have approximately 5 × 10^8^ cells/mL and 2.5 × 10^8^ cells/mL of *A. tumefaciens* and *E. africanus*, respectively, giving an MOI of 2:1. The co-culture was applied dropwise to sterile Whatman no. 5 qualitative filter papers placed on co-cultivation plates. The co-cultivation plates were incubated at 25°C for 2 days, then transferred to BHI agar plates containing 300 µg/mL hygromycin B and 200 μM cefotaxime. The plates were incubated at 37°C for 10–14 days until *E. africanus* transformant colonies appeared. Single colonies were picked and streaked on fresh BHI plates containing 300 µg/mL hygromycin B. From these plates, single colonies were used to inoculate BHI broth to grow liquid cultures of individual transformants. Glycerol stocks (20%) of liquid cultures were preserved at −80°C.

### Infection of macrophages with *E. africanus* and live-cell imaging

J774A.1 murine macrophage cells (ATCC: TIB-67) were seeded at a density of 2 × 10^5^ cells in 0.3 mL macrophage medium (DMEM high glucose GlutaMAX, 10% FBS, penicillin/streptomycin [Gibco]) in 8-well imaging slides (ibidi GMBH, cat. no. 80826) 24 h prior to the addition of *E. africanus* yeast. *E. africanus* yeast was grown for 3 days in BHI broth, then washed with PBS and counted using a haemocytometer. A total of 2 × 10^5^ yeast cells in 300 µL macrophage media were added to macrophages in imaging slides to achieve a ratio of one yeast cell per macrophage (MOI = 1). The slides were then set up for time-lapse imaging under the conditions of 37°C, 5% CO_2_. Imaging was started 1 h post-yeast addition. Imaging was done using a Zeiss AxioObserver fluorescence microscope at 40× magnification. Analysis of observed budding time was done by examining yeast cells that were intracellular or extracellular, at *t* = 0, with no visible buds.

### Mouse model of infection and flow cytometry

To examine the responses to *E. africanus* in the mouse lung, three groups of mice were analyzed (*n* = 6 per group): (i) uninfected mice, (ii) mice infected with the wild-type clinical isolate, and (iii) mice infected with the GFP-fluorescent *E. africanus* strain. Male C57BL/6 mice aged 8–12 weeks were obtained from the University of Cape Town Animal Research Specific Pathogen Free Facility. *E. africanus* wild-type and GFP-fluorescent strains were grown in BHI broth for 3 days at 37°C until OD600 reached approximately 1.0. Yeast cells were washed twice with PBS before counting using a haemocytometer. Mice were anaesthetized with ketamine and xylazine (80 mg/kg and 16 mg/kg, respectively), then infected intranasally with 50 µL of yeast cell suspension containing 1 × 10^7^ yeast cells. Animal welfare was monitored daily. After 7 days, mice were euthanized by halothane inhalation, and the lungs were removed and stored in DMEM + 5% FBS on ice. Lungs were digested in digestion buffer (15) containing 50 units/mL Collagenase Type I and 13 μg/mL DNAseI for 1 h at 37°C. The digested lungs were passed through a 70 µm cell strainer using a sterile syringe plunger. This process was repeated using a 40 µm cell strainer. Red blood cells were lysed using 1× RBC lysis buffer (eBioscience). The remaining cells were collected by centrifugation, resuspended in DMEM + 5% FBS, and then counted using a hemocytometer. A total of 2 × 10^6^ cells per well were added to a V-bottom 96-well plate. Myeloid cells were stained in MACS buffer (0.5% BSA, 2 mM EDTA in PBS) using the antibodies in Table 1, together with 1% Fc block and 2% heat-inactivated rat serum, for 20 min at 4°C in the dark, then washed twice with MACS buffer. 7-AAD (Biolegend) was used as the cell viability stain. Single-stained and unstained controls were used to compensate for spectral overlap. Stained cells were analyzed by flow cytometry using an LSR Fortessa (BD Immunocytometry Systems, San Jose, CA, USA) and BD FACS Diva software (v6.0). FlowJo software (v10.0.7) (Treestar, Ashland, OR, USA) was used for post-acquisition analysis and cell population determination. The gating strategy used is shown in Fig. 3A.

**TABLE 1:** Flow cytometry antibodies

Target (clone)	Conjugated fluorophore	Manufacturer (catalog number)	Dilution
CD45 (30-F11)	BV510	BD Biosciences (567800)	1:100
CD11b (M1/70)	BV421	BD Biosciences (566313)	1:100
CD11c (HL3)	Alexa Fluor 700	BD Biosciences (560583)	1:100
Ly-6G (1A8)	APC	BD Biosciences (560599)	1:100
F4/80 (BM8)	PE/Cy7	Biolegend (123113)	1:100
Siglec-F (E50-2440)	APC/Cy7	BD Biosciences (565527)	1:50
I-A/I-E (MHCII) (M5/114.15.2)	PE/Cy5	Biolegend (107611)	1:100

### Statistical analysis and software

Values are reported as means ± standard deviation. Differences between groups were determined using a two-tailed Student’s *t*-test or one-way ANOVA using GraphPad Prism v10.6.1. Plasmid map generation and alignments of DNA sequence data were done using SnapGene v8.02. ImageJ 1.54p was used for image analysis (mean fluorescence intensities and total cell counting).

### Plasmid construction

Plasmids pAG21, pAG22, pCR115 (18), pSL01, and pCR745 (6) were provided by C.A.R. Isolation of plasmid DNA, restriction enzyme cloning, and PCR/gel purification were done using standard methods, and plasmids were maintained in *E. coli* DH5α. Restriction enzymes and ligation kits were purchased from New England Biolabs. An episomal GFP expression plasmid (pHygR-GFP) was constructed by digestion of pAG22 with EcoRI and HindIII, and ligating the fragment containing the GFP and *hph* expression cassettes into the similarly digested pCR115 vector backbone. To construct pHyg-HcAGS1-RNAi (AGS1 inverted repeat) used for *AGS1* RNAi, the *GFP* sequence from plasmid pHygR-GFP was exchanged with the *AGS1* inverted repeat sequence from pCR115 by digestion with AscI and SpeI. A control *GFP* RNAi plasmid (pHygR-GFP-RNAi) was constructed by inserting an inverted repeat of *GFP* into pHygR-GFP by PCR with GFP-SpeI-F and XhoI-GFP-R primers and restriction enzyme cloning. For CRISPR/Cas9-mediated disruption of GFP, plasmid pCR115-sgGFP was created: a portion of plasmid pCR745 (6) containing the guide RNA targeting GFP was digested with SwaI and AvrII and ligated into similarly digested pSL01 (6), resulting in plasmid pSL01-sgGFP. For CRISPR/Cas9-mediated disruption of *URA5*, a synthetic DNA sequence containing the guide RNA sequence, hammerhead and HDV ribozyme sequences, and part of the tracr RNA sequence, including 15 bp upstream and downstream homology regions, was designed for cloning into pSL01 as described in reference 6. The guide RNA sequence was designed using CRISPOR (https://crispor.gi.ucsc.edu/) (34) to maximize the chance of indels while preventing off-target effects. This sequence was synthesized by Integrated DNA Technologies (Leuven, Belgium). The synthetic DNA sequence was cloned into SwaI-digested pSL01 and transformed into competent *E. coli* cells using In-Fusion HD Cloning Kit (Takara Bio) using the manufacturer’s recommendations. Table 2 summarizes the plasmids used in this study.

**TABLE 2:** Plasmids used in the study

Plasmid	Application	Selection
pAG22	ATMT	Hygromycin B (300 µg/mL)
pAG21	ATMT	G418 (1 mg/mL)
pCR115	*AGS1* RNAi	Uracil auxotrophy
pHygR-GFP	GFP episomal plasmid	Hygromycin B (300 µg/mL)
pHygR-GFP-RNAi	GFP inverted repeat plasmid (RNAi control)	Hygromycin B (300 µg/mL)
pHygR-HcAGS1-RNAi	*AGS1* RNAi	Hygromycin B (300 µg/mL)
pCR745	CRISPR/Cas9	Uracil auxotrophy
pSL01	CRISPR/Cas9	Hygromycin B (300 µg/mL)
pSL01-sgGFP	CRISPR/Cas9	Hygromycin B (300 µg/mL)
pSL01-sgURA	CRISPR/Cas9	Hygromycin B (300 µg/mL)

### Transformation of yeast with episomal plasmids by electroporation

Transformation of *E. africanus* with plasmids by electroporation was based on previously established methods for *H. capsulatum* (17). Briefly, *E. africanus* yeasts were grown in 5 mL BHI broth for 3 days until OD600 reached approximately 1.0. Yeast cells were collected by centrifugation and resuspended in 2 mL of 10% mannitol. Plasmids were linearized by digestion with PmeI to expose the telomeres, then column-purified and eluted with nuclease-free water. Three hundred microliters of *E. africanus* yeast suspension (1 × 10^7^ yeast cells) was mixed with 200 ng purified plasmid in a chilled electroporation cuvette (0.2 cm gap), then electroporated in a single pulse using a Bio-Rad Gene Pulser II system with the following settings: 1.25 kV, 50 mF, 600 Ω, which gave a time constant of 8–12 ms. Following electroporation, 500 µL of BHI broth was mixed with cells, and this cell suspension was applied dropwise to sterile Whatman no. 5 qualitative filter papers placed on BHI agar plates with no selection. These plates were incubated for 48 h at 37°C to allow for recovery of transformants, before transferring the membranes to BHI agar plates containing 300 µg/mL hygromycin B. The plates were incubated at 37°C for 10–14 days until transformant colonies appeared. Single colonies were picked and streaked onto fresh BHI plates (300 µg/mL hygromycin B). Selection with hygromycin B (300 µg/mL) was maintained for growing liquid cultures, after which −80°C glycerol stocks were prepared for long-term storage of strains, as described above.

### Cell wall α(1,3)-glucan staining of RNAi strains

Yeasts harboring the *AGS1* RNAi plasmid pHygR-HcAGS1-RNAi or the control plasmid pHygR-GFP-RNAi were grown for 3 days in BHI broth with 300 µg/mL hygromycin B until cell density reached an OD600 of 1.0. Cells from 300 µL of culture were collected by centrifugation, then washed twice with PBS, and fixed in 4% paraformaldehyde overnight at 4°C. Cells were washed twice with PBS, then blocked with 1% BSA in PBS for 1 h. Cells were then stained with 1:150 mouse IgM MOPC-104E antibody (M5170, Sigma-Aldrich) in 1% BSA, PBS for 2 h at ambient temperature. The cells were washed twice by centrifugation and resuspended in PBS and stained with 1:400 anti-mouse IgM secondary antibody conjugated to AlexaFluor488 (Invitrogen, A-21042) for 1 h at ambient temperature. To stain total yeast cells, calcofluor white was added at a final concentration of 100 µg/mL and incubated for 15 min at ambient temperature in the dark. Cells were washed twice with PBS and resuspended in 20 µL PBS. A 5 µL sample of stained cells was spotted onto a microscope slide, allowed to air-dry, and mounted with ProLong Glass Antifade Mountant (P36984, Invitrogen). The slides were examined using a Zeiss Axiovert 200M fluorescence microscope with a monochrome Zeiss AxioCam HRm camera at 100× magnification. Exposure times were kept constant for all samples when capturing images for mean fluorescence analysis. Fluorescence of the population was determined by measuring the background-corrected mean gray values for 10 fields of view each using ImageJ, for two pooled independent experiments, then dividing this value by the number of total cells (as determined by calcofluor white staining).

### Targeted mutagenesis using CRISPR/Cas9-plasmids

A GFP-expressing strain of *E. africanus* was generated through ATMT using pAG21 (equivalent to pAG22 but with a G418 resistance marker instead of hygromycin B resistance). The GFP-fluorescent strain was subsequently transformed with PmeI-linearized plasmid pSL01-sgGFP by electroporation. Hygromycin-resistant transformant colonies were picked and grown in BHI broth for successive passages (1:50 dilution), maintaining hygromycin selection every 3 days. Samples of these cultures were examined by fluorescence microscopy for GFP expression. For DNA sequencing, yeast cells from 1 mL of culture were collected by centrifugation, lysed with 200 µL of lysis buffer (2% Triton X-100, 20 mM EDTA), and beaten (2 min) with approximately 100 µL of glass beads (0.5 µm diameter). One hundred microliters of phenol and 100 µL of chloroform-isoamyl alcohol (25:24:1) were added, and the phases were separated by centrifugation. DNA was recovered by transferring 50 μL of the aqueous (upper) phase into 200 μL of distilled water. This crude DNA-containing solution was used as the template (2% vol/vol) for PCR using Taq polymerase and primers flanking the expected gene editing sites: GFP-SpeI-F and GFP-Xho-R, or Ura seq-F and Ura seq-R.

For mutation of *URA5* to create a uracil auxotroph, a guide RNA sequence targeting URA5 was designed using CRISPOR (https://crispor.gi.ucsc.edu/) (34). A DNA construct containing the crRNA, hammerhead and HDV ribozyme sequences, and part of the tracrRNA sequence, with 15 bp flanking homology arms, was synthesized as described in reference 6 for cloning into plasmid pSL01, by Integrated DNA Technologies (Leuven, Belgium). The sequence of this construct is shown in Table 3. Cloning of this synthetic DNA into SwaI-digested plasmid pSL01 was done through InFusion Cloning (Takara Biotec) as per the manufacturer’s recommendations. Constructs were verified through Sanger sequencing. *E. africanus* was transformed with this plasmid by electroporation as described above, followed by selection with hygromycin B. After four passages in liquid culture, cultures were streaked onto BHI plates supplemented with 1 mg/mL 5-fluoroorotic acid (5-FOA, R0811, ThermoFisher) and 100 µg/mL uracil to isolate uracil auxotrophs. Uracil auxotrophy was confirmed by the inability to grow in DMEM/F12 media (11320033, Gibco), which lacks uracil, compared to growth in parallel in DMEM/F12 media supplemented with 100 µg/mL uracil. Independent clones were picked for Sanger sequencing.

**TABLE 3:** Primers and oligonucleotides

Primer name/description	Sequence (5' to 3')
GFP-SpeIF	GAGAACTAGTATGAGCAAGGGCGAGGAGC
GFP-XhoIR	GAGACTCGAGCTTGTACAGCTCGTCCATGC
Synthesized sequence for URA5 CRISPR/Cas9 mutation^*a*^	CTCTCCCAATTTTCACATTTAAATCCCGCC ACCCGATGTCTGATGAGTCCGTGAGGAC GAAACGAGTAAGCTCGTCACATCGGGTT TCGGAATGGA GTTTTAGAGCTAGAAATAG CAAGTTAAAATAAGGCTAGTCCGTTAT
Ura seq F	TTGACACAGCCCATAAACG
Ura seq R	ATCGCCTTCTATATTCCTCC

^*a*^ Guide sequence underlined.

## Data Availability

All data that support the findings of this study, any plasmid(s) or its sequence files, and strains of microorganisms are available from the corresponding author upon reasonable request, provided that a suitable material transfer agreement is in place (in the case of biological samples).

## SUPPLEMENTAL MATERIAL

Supplemental figures msphere.00180-26-s0001.pdf Figures S1 and S2.

ASM does not own the copyrights to Supplemental Material that may be linked to, or accessed through, an article. The authors have granted ASM a non-exclusive, world-wide license to publish the Supplemental Material files. Please contact the corresponding author directly for reuse.

## References

[1] Dukik K, Muñoz JF, Jiang Y, Feng P, Sigler L, Stielow JB, Freeke J, Jamalian A, Gerrits van den Ende B, McEwen JG, Clay OK, Schwartz IS, Govender NP, Maphanga TG, Cuomo CA, Moreno LF, Kenyon C, Borman AM, de Hoog S. 2017. Novel taxa of thermally dimorphic systemic pathogens in the Ajellomycetaceae (Onygenales). Mycoses 60:296–309. doi:10.1111/myc.12601 28176377 PMC5775888

[2] Schwartz IS, Govender NP, Sigler L, Jiang Y, Maphanga TG, Toplis B, Botha A, Dukik K, Hoving JC, Muñoz JF, de Hoog S, Cuomo CA, Colebunders R, Kenyon C. 2019. Emergomyces: the global rise of new dimorphic fungal pathogens. PLoS Pathog 15:e1007977. doi:10.1371/journal.ppat.1007977 31536607 PMC6752945

[3] Schwartz IS, Kenyon C, Lehloenya R, Claasens S, Spengane Z, Prozesky H, Burton R, Parker A, Wasserman S, Meintjes G, Mendelson M, Taljaard J, Schneider JW, Beylis N, Maloba B, Govender NP, Colebunders R, Dlamini S. 2017. AIDS-related endemic mycoses in Western Cape, South Africa, and clinical mimics: a cross-sectional study of adults with advanced HIV and recent-onset, widespread skin lesions. Open Forum Infect Dis 4:ofx186. doi:10.1093/ofid/ofx186 29164168 PMC5695619

[4] Schwartz IS, McLoud JD, Berman D, Botha A, Lerm B, Colebunders R, Levetin E, Kenyon C. 2018. Molecular detection of airborne Emergomyces africanus, a thermally dimorphic fungal pathogen, in Cape Town, South Africa. PLoS Negl Trop Dis 12:e0006174. doi:10.1371/journal.pntd.0006174 29357352 PMC5800596

[5] Jiang Y, Dukik K, Muñoz JF, Sigler L, Schwartz IS, Govender NP, Kenyon C, Feng P, van den Ende BG, Stielow JB, Stchigel AM, Lu H, de Hoog S. 2018. Phylogeny, ecology and taxonomy of systemic pathogens and their relatives in Ajellomycetaceae (Onygenales): Blastomyces, Emergomyces, Emmonsia, Emmonsiellopsis. Fungal Divers 90:245–291. doi:10.1007/s13225-018-0403-y

[6] Rappleye CA. 2023. Targeted gene deletions in the dimorphic fungal pathogen Histoplasma using an optimized episomal CRISPR/Cas9 system. mSphere 8:e0017823. doi:10.1128/msphere.00178-23 37389430 PMC10449496

[7] Brechting PJ, Shah C, Rakotondraibe L, Shen Q, Rappleye CA. 2023. Histoplasma capsulatum requires peroxisomes for multiple virulence functions including siderophore biosynthesis. mBio 14:e0328422. doi:10.1128/mbio.03284-22 37432032 PMC10470777

[8] Kujoth GC, Sullivan TD, Klein BS. 2020. Gene editing in dimorphic fungi using CRISPR/Cas9. CP Microbiology 59:e132. doi:10.1002/cpmc.132 PMC7783865 33315302

[9] Kemski MM, Stevens B, Rappleye CA. 2013. Spectrum of T-DNA integrations for insertional mutagenesis of Histoplasma capsulatum. Fungal Biol 117:41–51. doi:10.1016/j.funbio.2012.11.004 23332832 PMC3552300

[10] Youseff BH, Rappleye CA. 2012. RNAi-based gene silencing using a GFP sentinel system in Histoplasma capsulatum*.* Methods Mol Biol 845:151–164. doi:10.1007/978-1-61779-539-8_10 22328373

[11] Sullivan TD, Rooney PJ, Klein BS. 2002. Agrobacterium tumefaciens integrates transfer DNA into single chromosomal sites of dimorphic fungi and yields homokaryotic progeny from multinucleate yeast. Eukaryot Cell 1:895–905. doi:10.1128/EC.1.6.895-905.2002 12477790 PMC138753

[12] Idnurm A, Bailey AM, Cairns TC, Elliott CE, Foster GD, Ianiri G, Jeon J. 2017. A silver bullet in a golden age of functional genomics: the impact of Agrobacterium-mediated transformation of fungi. Fungal Biol Biotechnol 4:6. doi:10.1186/s40694-017-0035-0 28955474 PMC5615635

[13] Sil A, Andrianopoulos A. 2014. Thermally dimorphic human fungal pathogens—polyphyletic pathogens with a convergent pathogenicity trait. Cold Spring Harb Perspect Med 5:a019794. doi:10.1101/cshperspect.a019794 25384771 PMC4526722

[14] Zemska O, Rappleye CA. 2012. Agrobacterium-mediated insertional mutagenesis in Histoplasma capsulatum. Methods Mol Biol 845:51–66. doi:10.1007/978-1-61779-539-8_4 22328367

[15] Höft MA, Duvenage L, Salie S, Keeton R, Botha A, Schwartz IS, Govender NP, Brown GD, Hoving JC. 2024. The pathogenesis of experimental emergomycosis in mice. PLoS Negl Trop Dis 18:e0011850. doi:10.1371/journal.pntd.0011850 38198478 PMC10805315

[16] Woods JP, Goldman WE. 1993. Autonomous replication of foreign DNA in Histoplasma capsulatum: role of native telomeric sequences. J Bacteriol 175:636–641. doi:10.1128/jb.175.3.636-641.1993 8423138 PMC196199

[17] Woods JP, Heinecke EL, Goldman WE. 1998. Electrotransformation and expression of bacterial genes encoding hygromycin phosphotransferase and beta-galactosidase in the pathogenic fungus Histoplasma capsulatum. Infect Immun 66:1697–1707. doi:10.1128/IAI.66.4.1697-1707.1998 9529100 PMC108107

[18] Rappleye CA, Engle JT, Goldman WE. 2004. RNA interference in Histoplasma capsulatum demonstrates a role for α-(1,3)-glucan in virulence. Mol Microbiol 53:153–165. doi:10.1111/j.1365-2958.2004.04131.x 15225311

[19] Ray SC, Rappleye CA. 2022. Mac1-dependent copper sensing promotes Histoplasma adaptation to the phagosome during adaptive immunity. mBio 13:e0377321. doi:10.1128/mbio.03773-21 35404120 PMC9040751

[20] Shen Q, Ray SC, Evans HM, Deepe GS, Rappleye CA. 2020. Metabolism of gluconeogenic substrates by an intracellular fungal pathogen circumvents nutritional limitations within macrophages. mBio 11:e02712-19. doi:10.1128/mBio.02712-19 32265333 PMC7157778

[21] Dade J, DuBois JC, Pasula R, Donnell AM, Caruso JA, Smulian AG, Deepe GS Jr. 2016. HcZrt2, a zinc responsive gene, is indispensable for the survival of Histoplasma capsulatum in vivo. Med Mycol 54:865–875. doi:10.1093/mmy/myw045 27335059 PMC5057459

[22] Hilty J, George Smulian A, Newman SL. 2011. Histoplasma capsulatum utilizes siderophores for intracellular iron acquisition in macrophages. Med Mycol 49:633–642. doi:10.3109/13693786.2011.558930 21341981

[23] Kujoth GC, Sullivan TD, Merkhofer R, Lee T-J, Wang H, Brandhorst T, Wüthrich M, Klein BS. 2018. CRISPR/Cas9-mediated gene disruption reveals the importance of zinc metabolism for fitness of the dimorphic fungal pathogen Blastomyces dermatitidis. mBio 9:e00412-18. doi:10.1128/mBio.00412-18 29615501 PMC5885028

[24] Joehnk B, Ali N, Voorhies M, Walcott K, Sil A. 2023. Recyclable CRISPR/Cas9-mediated gene disruption and deletions in Histoplasma. mSphere 8:e0037023. doi:10.1128/msphere.00370-23 37819140 PMC10732100

[25] Azimova D, Herrera N, Duvenage L, Voorhies M, Rodriguez RA, English BC, Hoving JC, Rosenberg O, Sil A. 2022. Cbp1, a fungal virulence factor under positive selection, forms an effector complex that drives macrophage lysis. PLoS Pathog 18:e1010417. doi:10.1371/journal.ppat.1010417 35731824 PMC9255746

[26] Allendoerfer R, Deepe GS Jr. 1997. Intrapulmonary response to Histoplasma capsulatum in gamma interferon knockout mice. Infect Immun 65:2564–2569. doi:10.1128/iai.65.7.2564-2569.1997 9199420 PMC175362

[27] Calich VLG, da Costa TA, Felonato M, Arruda C, Bernardino S, Loures FV, Ribeiro LRR, de Cássia Valente-Ferreira R, Pina A. 2008. Innate immunity to Paracoccidioides brasiliensis infection. Mycopathologia 165:223–236. doi:10.1007/s11046-007-9048-1 18777631

[28] Deepe GS, Gibbons RS, Smulian AG. 2008. Histoplasma capsulatum manifests preferential invasion of phagocytic subpopulations in murine lungs. J Leukoc Biol 84:669–678. doi:10.1189/jlb.0308154 18577715 PMC2516902

[29] Van Prooyen N, Henderson CA, Hocking Murray D, Sil A. 2016. CD103^+^ conventional dendritic cells are critical for TLR7/9-dependent host defense against Histoplasma capsulatum, an endemic fungal pathogen of humans. PLoS Pathog 12:e1005749. doi:10.1371/journal.ppat.1005749 27459510 PMC4961300

[30] Szymczak WA, Deepe GS Jr. 2010. Antigen-presenting dendritic cells rescue CD4-depleted CCR2^−/−^ mice from lethal Histoplasma capsulatum infection. Infect Immun 78:2125–2137. doi:10.1128/IAI.00065-10 20194586 PMC2863525

[31] Allen HL, Deepe GS Jr. 2005. Apoptosis modulates protective immunity to the pathogenic fungus Histoplasma capsulatum. J Clin Invest 115:2875–2885. doi:10.1172/JCI25365 16151533 PMC1199552

[32] Kügler S, Schurtz Sebghati T, Groppe Eissenberg L, Goldman WE. 2000. Phenotypic variation and intracellular parasitism by Histoplasma capsulatum. Proc Natl Acad Sci USA 97:8794–8798. doi:10.1073/pnas.97.16.8794 10922037 PMC34014

[33] Rice P, Longden I, Bleasby A. 2000. EMBOSS: the european molecular biology open software suite. Trends Genet 16:276–277. doi:10.1016/s0168-9525(00)02024-2 10827456

[34] Concordet JP, Haeussler M. 2018. CRISPOR: intuitive guide selection for CRISPR/Cas9 genome editing experiments and screens. Nucleic Acids Res 46:W242–W245. doi:10.1093/nar/gky354 29762716 PMC6030908

[35] Retallack DM, Heinecke EL, Gibbons R, Deepe GS, Woods JP. 1999. The URA5 gene is necessary for Histoplasma capsulatum growth during infection of mouse and human cells. Infect Immun 67:624–629. doi:10.1128/IAI.67.2.624-629.1999 9916068 PMC96364

[36] den Dulk-Ras A, Hooykaas PJ. 1995. Electroporation of Agrobacterium tumefaciens. Methods Mol Biol 55:63–72. doi:10.1385/0-89603-328-7:63 8528423

